# Increased Ratio of Matrix Metalloproteinase-9 (MMP-9)/Tissue Inhibitor Metalloproteinase-1 from Alveolar Macrophages in Chronic Asthma with a Fast Decline in FEV_1_ at 5-Year Follow-up

**DOI:** 10.3390/jcm8091451

**Published:** 2019-09-12

**Authors:** Fu-Tsai Chung, Hung-Yu Huang, Chun-Yu Lo, Yu-Chen Huang, Chang-Wei Lin, Chih-Chen He, Jung-Ru He, Te-Fang Sheng, Chun-Hua Wang

**Affiliations:** 1 Department of Thoracic Medicine, Chang Gung Memorial Hospital, Taipei 105, Taiwan; vikingchung@yahoo.com.tw (F.-T.C.) compaction71@gmail.com (H.-Y.H.); mixova@yahoo.com (C.-Y.L.); yuchenhahaha@gmail.com (Y.-C.H.); heh0229@yahoo.com.tw (C.-C.H.); mitosenorico@gmail.com (J.-R.H.); rubysheng050@gmail.com (T.-F.S.); 2 College of Medicine, Chang Gung University, Taoyuan 333, Taiwan; 3 Division of Pulmonary and Critical Care, Department of Internal Medicine, Saint Paul’s Hospital, Taoyuan 330, Taiwan; 4 Graduate Institute of Clinical Medical Sciences, College of Medicine, Chang Gung University, Taoyuan 333, Taiwan

**Keywords:** asthma, airway remodeling, matrix metalloproteinases-9, tissue inhibitor of metalloproteinase-1, alveolar macrophages

## Abstract

Chronic asthma is associated with progressive airway remodeling, which may contribute to declining lung function. An increase in matrix metalloproteinases-9 (MMP-9)/tissue inhibitor metalloproteinase-1 (TIMP-1) may indicate airway inflammation and bronchial injury. Bronchial biopsy specimens and alveolar macrophages (AMs) were obtained from patients with asthma under regular treatment with inhaled corticosteroids or combination therapy and normal subjects (*n* = 10). Asthmatics included those with a slow forced expiratory volume in one second (FEV_1_) decline (<30 mL/year, *n* = 13) and those with a fast FEV_1_ decline (≥30 mL/year, *n* = 8) in 5-year follow-up. Immunostaining expression of MMP-9 and TIMP-1 was detected in airway tissues. MMP-9 and TIMP-1 was measured from AMs cultured for 24 h. After the 5-year treatment, the methacholine airway hyperresponsiveness of the slow FEV_1_ decline group was decreased, but that of the fast FEV_1_ decline group was increased (PC_20_, provocative concentration causing a 20% decrease in FEV_1_, 3.12 ± 1.10 to 1.14 ± 0.34 mg/dL, *p* < 0.05). AMs of asthma with a fast FEV_1_ decline released a higher level of MMP-9 (8.52 ± 3.53 pg/mL, *p* < 0.05) than those of a slow FEV_1_ decline (0.99 ± 0.20 pg/mL). The MMP-9/TIMP ratio in the fast FEV_1_ decline group (0.089 ± 0.032) was higher than that of the slow FEV_1_ decline group (0.007 ± 0.001, *p* < 0.01). The annual FEV_1_ decline in 5 years was proportional to the level of MMP-9 (r = 57, *p* < 0.01) and MMP-9/TIMP-1 ratio (r = 0.58, *p* < 0.01). The airways of asthma with greater yearly decline in FEV_1_ showed an increased thickness of submucosa and strong expression of MMP-9. An increase in MMP-9 and MMP-9/TIMP-1 in airways or AMs could be indicators of chronic airway inflammation and contribute to a greater decline in lung function of patients with chronic asthma.

## 1. Introduction

Asthma is a chronic respiratory disease of airway inflammation that manifests as variable airflow limitation [1]. Patients with asthmatic airway inflammation develop tissue injury with subsequent structural changes, which is termed airway wall remodeling [2,3]. Elderly or longer duration of asthma is correlated to airway wall remodeling and contributes to a decline in lung function [4]. The features of airway remodeling include smooth muscle hypertrophy, goblet-cell hyperplasia, subepithelial fibrosis, inflammatory cell infiltration, as well as epithelial shedding [5]. 

Matrix metalloproteinases (MMPs) are a family of enzymes that can break down proteins of the extracellular matrix (ECM), thus contributing to pathological processes of inflammation, wound healing, and fibrosis [6]. The MMPs also play an important role in several lung or airway diseases, or even lung cancer [7,8,9,10]. Additionally, increased levels of MMP-9 in serum, sputum, or lavage fluid were observed in patients with asthma [11,12,13]. MMP-9-deficient animals could inhibit airway inflammation and the immunoreactivity of MMP-9 has also been reported to be correlated with asthma severity [14]. Nevertheless, a defensive function of MMP-9 in asthma was reported through a heightened inflammation in MMP9-deficient mice [15,16]. Taken together, MMP-9 may be an important factor involved in asthma, but the production of MMP-9 in chronic asthma with persistent airway obstruction is still undetermined. 

The tissue inhibitors of matrix metalloproteinase (TIMPs) inhibit enzymatic activity of MMPs through binding to the MMPs [17,18]. The secretion of TIMP-1 is associated with MMP-9. TIMP-1 may possibly result in the thickening process of the basement membrane in asthma [19]. Therefore, MMP and TIMP imbalance may cause clinical differences in chronic airway diseases [20,21]. The ratio of MMP-9/TIMP-1 in the sputum of asthmatic patients has been shown to decrease after recovery from an acute exacerbation of asthma, which may imply that MMP9/TIMP has a negative correlation [22]. Through increasing the thickness of the airway wall by collagen deposition, a decreased ratio of MMP-9/TIMP-1 in chronic asthma may result in airway obstruction [4]. Vignola et al. [21] reported that the ratio of sputum MMP-9/TIMP-1 has a positive correlation with the forced expiratory volume in one second (FEV_1_) of asthmatic patients. Additionally, a previous report [23] showed that a low serum MMP-9/TIMP-1 ratio was found in asthmatic patients who show little FEV_1_ improvement with the treatment of corticosteroids. Despite some reports [22,23], which revealed the possibly negative correlation of MMP9, TIMP, and lung function in asthmatic patients, the relation among these parameters remains controversial. Some reports also presented no correlation between MMP9, TIMP, and lung functions [24], or positive correlations among MMP9, TIMP, and lung functions [25,26].

Our aim was to evaluate whether the ratio of MMP-9/TIMP-1 released from cultured alveolar macrophages was higher in chronic asthmatic patients who had a fast, yearly decline in FEV_1_. We also investigate whether the ratio of MMP-9/TIMP-1 was correlated to the magnitude of yearly FEV_1_ decline. 

## 2. Materials and Methods

### 2.1. Patient Population

Non-smoking asthmatic patients, aged 18 to 65 years, were recruited from outpatient clinics of the Chang Gung Memorial Hospital. Asthma was defined according to the American Thoracic Society criteria [27]. All asthmatic subjects had a >12% improvement in forced expiratory volume in one second (FEV_1_) with inhaled albuterol (400 μg) and bronchial hyperreactivity to methacholine (provocative concentration (PC) causing a 20% decrease in FEV_1_, PC_20_ < 8 mg/L). These patients received anti-asthma medications, which included inhaled and/or oral corticosteroids, inhaled long-acting β_2_-agonist, or a combination of these, and had been followed up at our clinics for more than 5 years. All of the patients with asthma had used inhaled corticosteroids all the time. All patients had been stable for at least 3 months and were taking their usual medications before entry into the study. Inhaled β_2_-agonists were withheld for 12 hours before methacholine testing.

The asthmatic subjects were divided into 2 groups: 13 asthmatic subjects (7 women and 6 men, aged 49.2 ± 3.4 years) with a slow FEV_1_ decline (< 30 mL/year) in the 5-year follow-up, and 8 asthmatic subjects (3 women and 5 men, aged 49.4 ± 3.7 years) with a fast FEV_1_ (≥ 30 mL/year) in the 5-year follow-up, as previously described [28]. During the following years, one of the asthmatics with a slow FEV_1_ decline and 4 asthmatics with a fast FEV_1_ decline experienced difficulty in controlling their asthma symptoms despite taking the maximum recommended dose of inhaled corticosteroids and inhaled long-acting β_2_-agonist. These patients needed additional therapy with long-term oral corticosteroids or a long-acting muscarinic antagonist. The calculation of annual FEV_1_ decline (mL/year) was determined by subtracting FEV_1_ at the first year by the second measurement 5 years later, then dividing this by 5 [28].

Ten normal non-smoking subjects (6 women and 4 men, aged 47.3 ± 2.8 years), who had normal pulmonary function and no evidence of bronchial hyperresponsiveness, allergic rhinitis, or asthma, were recruited. Their total IgE levels and eosinophil counts were normal and their serum-specific IgE was negative. 

### 2.2. Study Protocol 

All patients who satisfied the enrollment criteria performed routine pulmonary function tests and methacholine provocation testing after abstaining from short-acting oral or metered-dose inhaler bronchodilator use for 6 h, and long-acting β_2_ agonist for 24–48 hours. Asthmatic subjects underwent the procedure of fiberoptic bronchoscopy with bronchial biopsy and bronchoalveolar lavage. Informed consent was obtained from all patients before entry into the study. The study was approved by the Chang Gung Memorial Hospital Ethics Committee (IRB number: 90-13).

### 2.3. Fiberoptic Bronchoscopy 

All study patients received fiberoptic bronchoscopy under sedation. The procedure of bronchoscopy under sedation and administering of local anesthesia was performed in the study institution. Sedation with intravenous midazolam (5–10 mg), and local anesthesia with 2% xylocaine solution was performed during bronchoscopy. Oxygen saturation, blood pressure, and electrocardiography (ECG) were monitored during bronchoscopy. The bronchoscope was advanced through the nose and larynx, and then into the tracheal and bronchial lumen; bronchial biopsies were taken from the 4th or 5th subsegmental bronchus. The specimens were fixed by 4% paraformaldehyde for immunocytochemistry. 

### 2.4. Preparation of BAL Cells 

Bronchoalveolar lavage (BAL) was completed in subjects using 300 mL of 0.9% saline solution [29]. Sterile saline solution was instilled into the right fourth or fifth subsegmental bronchus. The lavage fluid was retrieved by gentle aspiration, collected, and filtered through two layers of sterile gauze. BAL fluid was kept on ice throughout processing. The collected BAL fluid was centrifuged at 600× *g* for 20 min at 4 °C. The cell pellet was obtained after centrifugation followed by consecutive washes and finally resuspended at 10^6^ cells per mL in RPMI-1640 (GIBCO, Grand Island, New York, NY, USA) containing 5% heat-inactivated fetal calf serum (FCS, Flow Laboratories, Paisley, Scotland, UK). Trypan blue exclusion was used to determine cell viability. Differential cell counts were determined by counting 500 cells on cytocentrifuge preparations using a modified Wright–Giemsa stain. BAL fluids were stored at −70 °C until analysis. The purified alveolar macrophages were placed in 6-well plates at 10^6^ cells/mL for 24 h at 37 °C and 5% CO_2_. The culture supernatant was collected and frozen at −70 °C until use. 

### 2.5. Immunocytochemistry 

**Figure 1:**
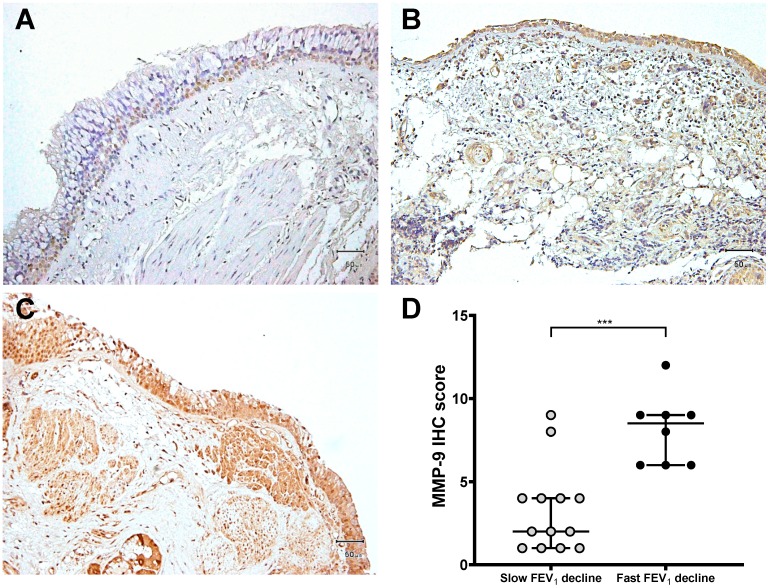
Immunohistochemical expression levels of MMP-9. An avidin–biotin complex immunohistochemical study for MMP-9 labeling was performed in airway tissues obtained from asthmatic patients with a slow FEV_1_ decline (A) and a fast FEV_1_ decline (B and C). Positive staining was defined as brown–yellow particles or tan–brown particles in the cytoplasm (magnification, 200×). (**A**) Airway tissue with weak staining (score 1); (**B**) airway tissue with moderate staining (score 6); (**C**) airway tissue with strong staining (score 12); (**D**) the immunohistochemistry score of MMP-9 was determined through a semi-quantitative assessment by calculating the intensity and percentage of positive cells. The central horizontal lines indicate the median, and the error bars (upper and lower horizontal lines) are the 75th percentile and 25th percentile, respectively, *** *p* < 0.0001. IHC, immunohistochemistry; MMP-9, matrix metalloproteinase-9.

**Figure 2:**
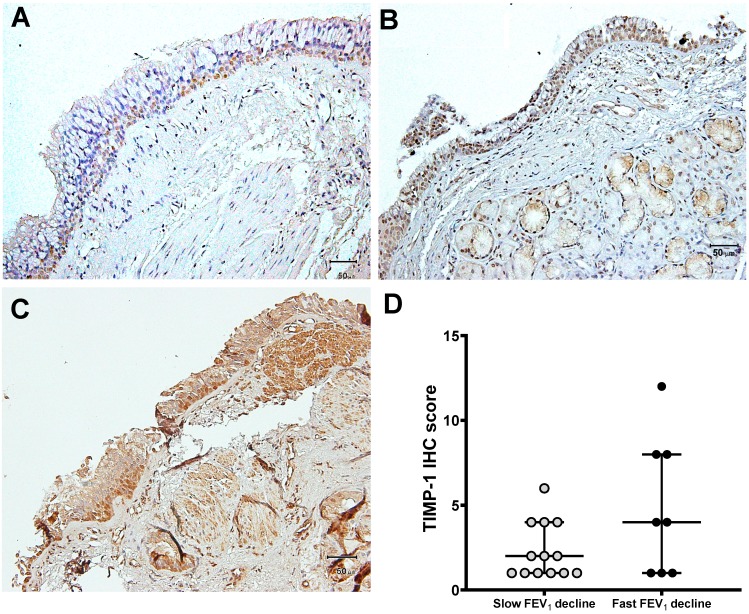
Immunohistochemical expression levels of TIMP-1. An avidin–biotin complex immunohistochemical study for TIMP-1 expression was performed in airway tissues derived from asthmatic patients with a slow FEV_1_ decline (A and B) and a rapid FEV_1_ decline (C). Positive staining was defined as brown–yellow particles or tan–brown particles in the cytoplasm (magnification, 200×). (**A**) airway tissue with weak staining (score 1); (**B**) airway tissue with moderate staining (score 6); (**C**) airway tissue with strong staining (score 12); (**D**) immunostaining of TIMP-1 was scored through a semi-quantitative assessment by calculating the intensity and percentage of positive cells. The central horizontal lines indicate the median, and the error bars (upper and lower horizontal lines) are the 75th percentile and 25th percentile, respectively. IHC, immunohistochemistry; TIMP-1, tissue inhibitor of matrix metalloproteinase-1.

Immunoreactivity for tissues was performed with the use of the avidin–biotin peroxidase complex method. Tissue sections (5 μm) from asthmatic subjects were incubated overnight at 4 °C with a variety of primary antibodies, including anti-human MMP-9 (Oncogen Science Inc, Cambridge, MA, USA) and TIMP-1 antibodies (Fuji Pharmaceutical Co, Toyama, Japan) [30]. Mouse immunoglobulin G1 (Dako, Kyoto, Japan) was used for negative controls. After washing in PBS/Tween 20 twice, the slides were counterstained by hematoxylin. Positive immunostaining was visualized as brown granules contained in the cytoplasm. The scores corresponding to MMP-9 and TIMP-1 immunostaining expression were evaluated by a semi-quantitative assessment using the intensity and percentage of positively stained cells, such as epithelial cells, inflammatory cells, and mucus gland or smooth muscle. The intensity of MMP-9 and TIMP-1 staining was scored as follows: 1, weak; 2, moderate; and 3, strong (Figure 1 and Figure 2). The percentage scores were determined by the following definition: 1, ≤25%; 2, 26–50%; 3, 51–75%; and 4, >75%. These scores were multiplied by the intensity and the percentage score. The range was between 1 and 12.

### 2.6. Hematoxylin and Eosin Staining (H and E)

The thickness of the epithelium, basement membrane, and subepithelial layer was investigated by H and E staining for 20 min at room temperature. The results were visualized using an Olympus BX51 microscope (Olympus Corporation, Tokyo, Japan).

### 2.7. MMP-9 and TIMP-1 ELISA 

Quantitative sandwich-type enzyme-linked immunoassay techniques (ELISA) were used to assay the secretory products in macrophage supernatants [26]. MMP-9 and TIMP-1 kits were used (Amersham Life Sciences, Arlington Heights, IL, USA) according to the manufacturer’s instructions. The optical density was measured with a spectrophotometer set to 450 mM for all assays. Quantification was performed by interpolation from a standard curve. 

### 2.8. Statistical Analysis

Data were presented as mean ± SEM. The data were analyzed using a Student’s *t*-test for paired or unpaired data. For data with even or uneven or variation, a Mann–Whitney U test or Wilcoxon signed rank test was used for unpaired or paired data, respectively. ANOVA with post hoc analysis was used when comparing data from three groups; *p* < 0.05 was considered significant.

## 3. Results

### 3.1. Demographic Features of Patients

**Figure 3:**
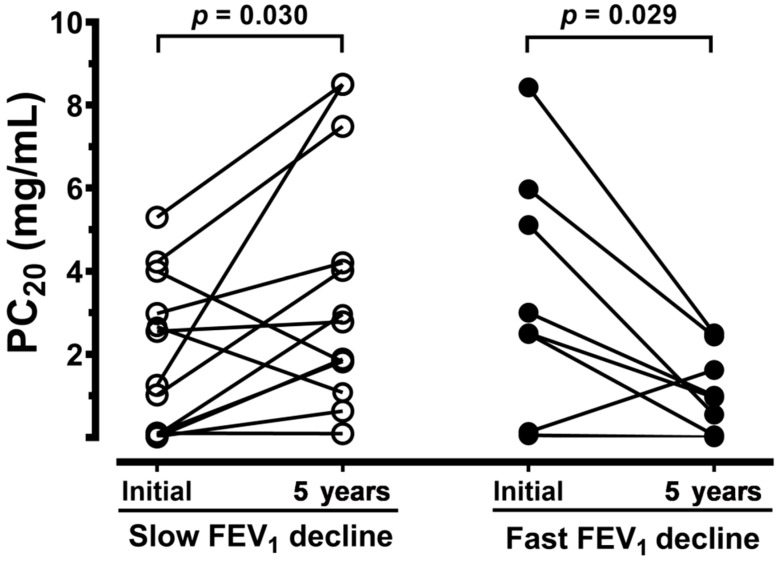
The individual concentration of methacholine provocation in asthmatic patients who had a slow FEV_1_ decline (open circle, *n* = 13) or a fast FEV_1_ decline (solid circle, *n* = 8) after receiving 5 years of inhaled anti-asthma medicine, compared with the initial values (initial). The significance is indicated.

Table 1 summarizes pulmonary function tests on asthmatic patients and normal subjects. The FVC, FEV_1_, and levels of PC_20_ in methacholine tests demonstrated no difference between the two groups of asthmatics. Initially, the PC_20_ was similar in both asthmatic groups. After 5 years, airway hyperresponsiveness of patients with a slow FEV_1_ decline was significantly decreased (PC_20_ from 1.87 ± 0.52 to 3.52 ± 0.81 mg/dL, *n* = 13, *p* < 0.05), while that of asthmatics with a fast FEV_1_ decline demonstrated an increased airway hyperresponsiveness (PC_20_ from 3.46 ± 1.02 to 1.14 ± 0.34 mg/dL, *n* = 8, *p* < 0.05) (Figure 3).

### 3.2. Cellular Profile Analysis of Bronchoalveolar Lavage

**Figure 4:**
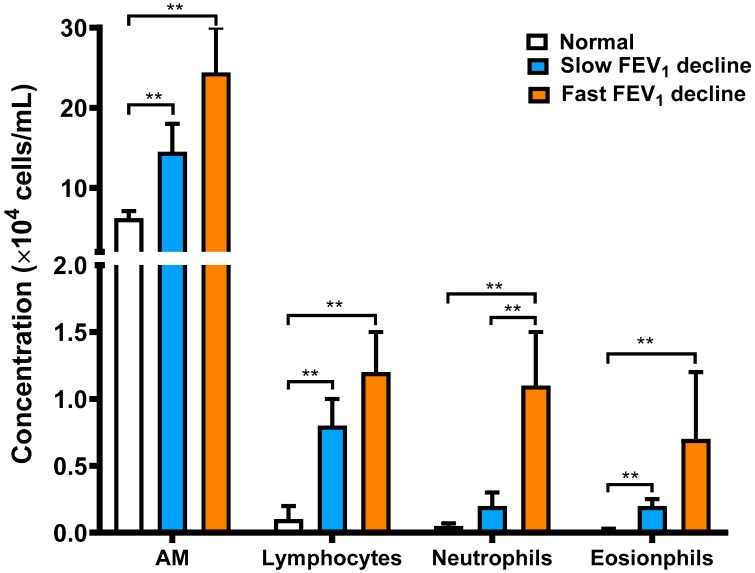
The concentration of different cell types in bronchoalveolar lavage fluid derived from normal subjects (Normal, *n* = 10), and asthmatic patients with a slow FEV_1_ decline (Slow FEV_1_ decline, *n* = 13) or a fast FEV_1_ decline (Fast FEV_1_ decline, *n* = 8); ** *p* < 0.01 is indicated.

The cellularity of lavage fluid was similar between the asthmatic groups but was significantly lower in normal subjects. Compared to normal subjects, there was an increase in the percentage of eosinophils and lymphocytes and a corresponding decrease in the proportion of alveolar macrophages in asthmatics with either a fast or a slow decline in FEV_1_ (Table 2). The cellularity of different cell types in normal subjects and asthmatics is presented in Figure 4 and had the same result as the percentage of cell types. The asthmatic patients with a fast FEV_1_ decline over 5 years had a marked rise in the proportion of neutrophils compared with those who exhibited a slow decline in FEV_1_ or normal subjects (Table 2). In addition, the magnitude of the annual FEV_1_ decline in asthmatics was highly correlated with the cellularity of neutrophils in BAL (r = 0.718, *n* = 21, *p* = 0.0002).

### 3.3. Expression of MMP-9 and TIMP-1 in Bronchial Biopsies

MMP-9 was expressed in airway tissue obtained from asthmatics, especially in epithelial cells, inflammatory cells, or gland cells (Figure 1A–C). Furthermore, asthmatic patients with a rapid FEV_1_ decline had a significantly higher immunohistochemistry (IHC) score of MMP-9 (median, 8.5; IQR, 6.5–9.0), when compared with asthmatic patients with a slow FEV_1_ decline (median, 2.0; IQR, 1.0–4.0) (*p* < 0.0001, Figure 1D). The IHC score of TIMP-1 expression in the airway showed no significant difference between the two groups (Figure 2D). The thickness of the basement membrane (15.5 ± 2.2 μm, *n* = 8, *p* = 0.0002) and subepithelial layer (138.3 ± 12.2 μm, *n* = 8, *p* < 0.0001) in asthmatics with a fast FEV_1_ decline was significantly increased (Table 3). However, the asthmatics with a slow FEV_1_ decline had a thinner basement membrane and subepithelial layer. 

### 3.4. Generation of MMP-9 and TIMP-1 From Macrophages

**Figure 5:**
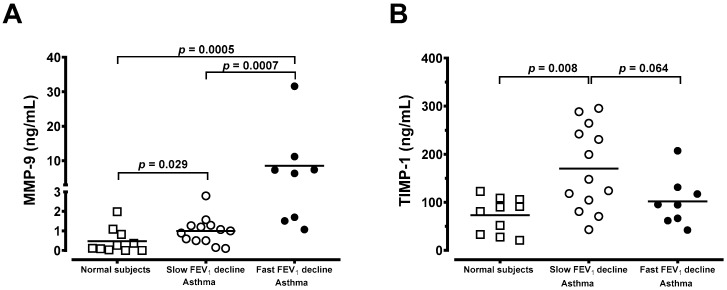
Individual concentration of (**A**) MMP-9 and (**B**) TIMP-1 spontaneously released from alveolar macrophages after 24 h culture in normal subjects (open square, *n* = 10), chronic asthma with a slow FEV_1_ decline (open circle, *n* = 13), and chronic asthma with a fast FEV_1_ decline in (solid circle, *n* = 8). The significance is indicated.

**Figure 6:**
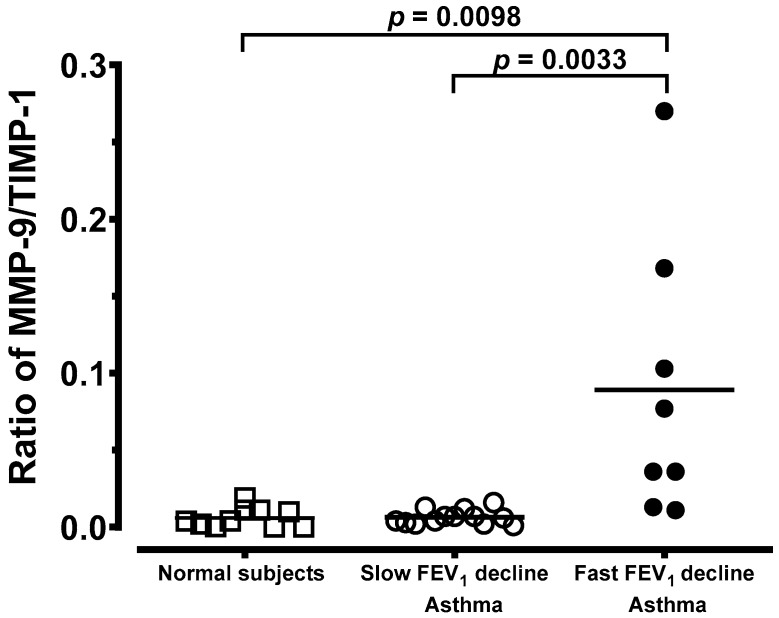
Ratio of MMP-9 to TIMP-1. Data are expressed as the individual value of MMP-9 to TIMP-1 ratio in normal subjects (open square, *n* = 10), chronic asthma with a slow FEV_1_ decline (open circle, *n* = 13), and chronic asthma with a fast FEV_1_ decline (solid circle, *n* = 8). The *p* values are presented.

Most importantly, alveolar macrophages (AMs) from chronic asthma with a fast FEV_1_ decline spontaneously released a higher amount of MMP-9 (8.52 ± 3.53 ng/mL, *n* = 8, *p* < 0.05) than those of asthma with a slow FEV_1_ decline (0.99 ± 0.20 ng/mL, *n* = 13) or normal subjects (0.47 ± 0.20 ng/mL, *n* = 10). The level of MMP-9 was significantly higher in asthma with a slow FEV_1_ decline compared to normal subjects (Figure 5A). Compared with MMP-9, concentrations of the inhibitor, TIMP-1, were higher in supernatants from normal subjects and asthmatic groups (Figure 5B). A significantly higher level of TIMP-1 released from AMs into the culture medium in asthmatics with a slow FEV_1_ decline over 5 years was observed when compared to that of asthmatics with a fast decline in FEV_1_ or normal subjects (Figure 5B). When data are expressed as the molar ratio of enzyme to inhibitor, chronic asthmatics with a fast FEV_1_ decline in 5 years had a significant increase in this ratio in AMs (Figure 6). The ratio of MMP-9/TIMP-1 showed no difference between the normal subjects and those suffering from chronic asthma with a slow FEV_1_ decline at the 5-year follow-up (Figure 6).

**Figure 7:**
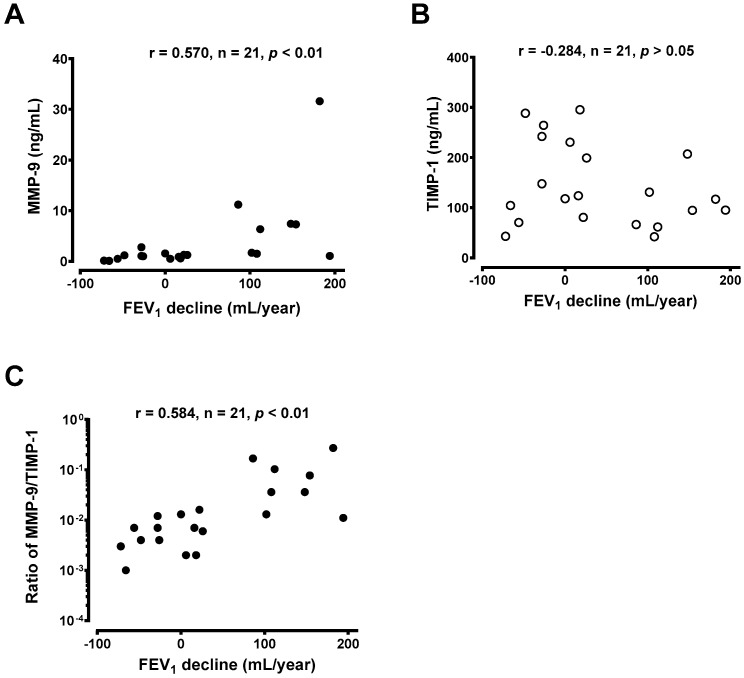
Correlation of the level of (**A**) MMP-9 and (**B**) TIMP-1 released from alveolar macrophages (AMs) cultured for 24 h with the magnitude of FEV_1_ decline per year (mL/year) from patients with chronic asthma receiving inhaled corticosteroids. (**C**) Correlation of the MMP-9/TIMP-1 ratio in 24 h AM culture with the magnitude of FEV_1_ decline per year over 5 years from patients with chronic asthma receiving inhaled corticosteroids.

The generation of MMP-9 released from AMs was positively associated with the annual decline in FEV_1_ (r = 570, *n* = 21, *p* < 0.01) (Figure 7A). However, there was no correlation between the level of TIMP-1 and the annual decline in FEV_1_ (Figure 6B). When the MMP-9/TIMP-1 ratios were plotted against the magnitude of FEV_1_ change, the annual decline in FEV_1_ was significantly proportional to the ratio of MMP-9/TIMP-1 (r = 0.584, *n* = 21, *p* < 0.01) (Figure 7C). 

## 4. Discussion

We demonstrated that the levels of MMP-9 expression in the epithelium, inflammatory cells, and submucosa as determined from immunostaining showed upregulation in asthmatic patients who regularly received inhaled corticosteroids and who had a rapid decline in pulmonary function at 5-year follow-up. The alveolar macrophages from these unstable asthmatics also spontaneously released higher amounts of MMP-9. Most importantly, the MMP-9 level and MMP-9/TIMP-1 ratio produced from AMs were significantly decreased in chronic asthma with a slow FEV_1_ decline. The higher levels of TIMP-1 released from cultured AM were observed in clinically stable asthmatics. The magnitude of annual FEV_1_ decline was proportional to the MMP-9 generation and the ratio of MMP-9/TIMP-1 released from alveolar macrophages, even though the patients had regularly received inhaled corticosteroids. An increased thickness of the basement membrane and subepithelial layer was observed in asthmatics with a fast FEV_1_ decline. Taken together, MMPs (mainly MMP-9) and TIMP-1 may contribute to progressive loss of lung function and increased cellular matrix fibrosis of the airway wall in chronic asthmatics who demonstrated a poor response to anti-asthma treatments. 

We reported that the generation of MMP-9 from alveolar macrophages and the ratio of MMP-9/TIMP-1 are strongly associated with the magnitude of FEV_1_ decline in chronic asthma, which is in agreement with the data of Vignola et al. [21]. The same authors have stressed the potential importance of MMP-9 and TIMP-1 imbalance in asthma by showing that the basal airway caliber was related to the ratio of MMP-9 to TIMP-1. It was reported that concentrations of MMP-9 in airway neutrophils and BAL fluid revealed a significant correlation after allergen challenge [31]. We also observed that the BAL fluid of asthmatics with a rapid decline in FEV_1_ exhibited a higher amount of neutrophils, while that of stable asthmatics did not show an increased amount of neutrophils. The cellularity of neutrophils in BAL was highly correlated to the annual FEV_1_ decline in asthmatics. These observations raise the possibility that neutrophils may cause injury to the airway and result in further remodeling of chronic unstable asthma, as has been suggested by studies showing persistent bronchial neutrophilia in severe asthma and status asthmatics [32]. Our results strongly suggest that alveolar macrophages and neutrophils may be important sources of MMP-9 release, leading some asthmatic patients—who are poorly responsive to inhaled corticosteroid treatment—to develop progressive irreversible airway scarring and fibrosis. Accordingly, an excess of MMP-9 release in the airway was associated with impairment in the lung function of FEV_1_. 

It is thought that the imbalance between MMPs and TIMPs may play an important role in the process of the degradation and synthesis of the extracellular matrix of the airway. Hoshino et al. [33] reported that deposition of the basement membrane matrix components—including collagen III, collagen V, and tenascin—in asthma correlated to the upregulated expression of MMP-9 and, therefore, airway remodeling in asthma could cause airflow obstruction and airway hyperresponsiveness. As well as the consuming mechanisms of the extracellular matrix, MMP-9 may modulate cytokines and other proteases [34]. MMP-9 may degrade alpha1-antitrypsin and preserve neutrophil elastase activity [35], thus activating the function of fibroblasts [36]. In addition, the binding of MMP-9 to CD44 can result in the release of TGF-β1 and, therefore, regulate extracellular matrix remodeling via fibroblast activation [37]. MMP-9 may also potentiate angiogenesis by vascular endothelial growth factor activation [34,38] and increase the production of angiostatin [39]. Our results showed increased thickness in the submucosa and basement membrane, as well as higher expression of MMP-9 in chronic asthma with rapid pulmonary function decline. The asthmatics with a fast FEV_1_ decline presented with higher airway hyperresponsiveness. Therefore, macrophages from these unstable asthmatics may release more MMP-9, leading to collagen deposition or neovascularization in the basement membrane of airways and contributing to airway hyperresponsiveness.

The TIMPs could bind to MMPs and inhibit their enzymatic activity. In our study, alveolar macrophages from chronic stable asthma patients responsive to inhaled corticosteroids released higher amounts of TIMP-1 than those of chronic unstable asthma patients who do not respond to inhaled corticosteroids or healthy subjects. Hoshino and colleagues [27] found that corticosteroid treatment can decrease the deposition of subepithelial collagen through downregulation of MMP-9 and upregulation of TIMP-1 in asthma. In addition, a higher level of TIMP in stable asthma potentially helps the airways to mitigate the degrading activities of MMPs [40]. Nevertheless, this process may restrict cell trafficking and tissue repair, and may cause increased deposition of the extracellular matrix through in vivo inhibition of MMP-9 or other MMPs. Russell et al. reported that the release and activity of MMP-9 and TIMP-1 by alveolar macrophages from patients with chronic obstructive pulmonary disease might be important in the development of COPD because these cells exhibit increased levels of MMP-9 elastolytic activity [41]. Meanwhile, Russell et al. also reported that dexamethasone prevented the increase in MMP-9 release, and increased TIMP-1 release. Similarly, alveolar macrophages released a higher amount of MMP-9 in chronic unstable asthma patients than those with stable asthma or normal subjects in our study (Figure 5 and Figure 6). Asthmatic patients had higher cell counts of BAL, including total cell counts, macrophage, lymphocytes, neutrophils, and eosinophils than those observed in normal subjects in Table 2 and Figure 4. MMP-9 is known to be produced by several inflammatory or structural cells, including bronchial epithelial cells, eosinophils, mast cells, and alveolar macrophages, and these may have a greater contribution relative to neutrophils in chronic asthma. This means that persistent airway or lung inflammation of chronic unstable asthmatics may be less responsive to anti-asthma treatment, which may contribute to higher cellularity of inflammatory cells, thus leading to the increased amount of MMP-9.

Although our patients have received inhaled corticosteroids for more than 5 years, some of these patients still demonstrated a progressive decline in pulmonary function and persistent airway structure change. A report showed that a high dose of inhaled corticosteroids did not change the levels of MMP-1, MMP-9, and TIMP-1 in induced sputum [42], although other authors have described a significant decrease in MMP-9 in the bronchial biopsies of asthmatics treated with inhaled corticosteroids [27]. Our results revealed that inhaled corticosteroids do not modify the expression of MMP-9 and TIMP-1 in AMs in patients with chronic asthma exhibiting a rapid decline in FEV_1_. Thus, inhaled corticosteroids cannot inhibit the airway remodeling of chronic asthma. Several reports have shown that neutrophil levels may increase in the airways in severe asthmatics or in patients with chronic asthma who exhibit a poor response to inhaled steroids [32,43]. Persistent generation of inflammatory products and their inhibitors in chronic asthma may cause airway injury and remodeling. The mechanisms should be further studied. 

Previous reports revealed that MMP-9 increases in severe asthma [40,44], and that it may be the cause of airflow obstruction through the induction of airway structural changes [45]. The persistently high levels of MMP-9 indicate that the remodeling of airways may be initiated at the beginning of asthma development and may be expressed as severe asthma. 

Extensive studies have revealed the role of MMPs in the pathogenesis of asthma, airway hyperresponsiveness (AHR), and asthma-associated airway remodeling [45,46]. MMP-9 was the first MMP to be investigated in depth for its role in the development of asthmatic pathology and was also the most highly expressed MMP in the BAL fluid and sputum of asthma patients [21,47]. Moreover, the high level of MMP-9 expression in bronchial biopsies from asthmatic patients [48] was associated with asthma severity [49] as well as the number of macrophages and neutrophils [50]. MMP-9 can degrade elastin and type IV collagen [51], an important component of the basement membrane and, thus, MMP-9 plays a role in the disruption of the basement membrane and contributes to increased ECM deposition, subepithelial fibrosis, and airway wall thickness [52]. Airway remodeling related to the thickness of the sub-basement membrane [45] has been linked to airway AHR and leads to the subsequent development of fixed airflow limitation and to a long-term decline in lung function in asthmatics [53]. Our recent study revealed that AMs produced excessive MMP-9 over TIMP-1, which was associated with increased AHR and was a predictor of the development of accelerated lung function decline [20]. Other MMPs, including MMP-1, -2, -3, or -12, are also studied as being involved in asthma [6]. MMP-1 is activated by mast cell tryptase, leading to a proproliferative extracellular matrix. The interactions of airway smooth muscle/mast cell contribute to asthma severity by transiently increasing MMP activation, thus inducing airway smooth muscle hyperplasia and AHR [54]. The potentially pro-remodeling roles for MMP-1 are involved in the promotion of airway smooth muscle proliferation. In stable mild-to-moderate asthma, MMP-2 in association with MMP-3 is released from bronchial fibroblasts and may have a negative effect on lung function and AHR [55]. However, studies on MMP-9 and MMP-2 double-knockout mice revealed that MMP-9, and not MMP-2, is the dominant airway MMP controlling inflammatory cell egression [56]. Although MMP-12 is suggested to be involved in asthma, this conclusion is based on studies in which mostly animal models are used [6]. In humans, the MMP-12-mediated pathological degradation of the ECM is associated with COPD patients [57]. Our study aimed to investigate whether the MMP released from AM was associated with AHR and increased expression in airways, thus contributing to subepithelial thickness and accelerated lung function decline. Therefore, our study focused on MMP-9. Nevertheless, a greater understanding of the involvement of MMPs in airway remodeling in asthma is indispensable and, thus, further studies are needed to examine the expression of other MMPs in airways and BAL fluid as well as their release from AMs. 

### Limitations

Alveolar macrophages are the predominant immune cells in the lung and are represented by the classically activated (or M1) and the alternatively activated (or M2) phenotypes according to their function [58]. Increased polarization and activation of M2 macrophages, which was induced by interleukin (IL)-4 and IL-13, are found in asthma and are suggested to be involved in asthma pathogenesis [59,60]. A previous study has shown that activation of M1 or M2 macrophages may upregulate a distinct group of MMPs and TIMP-3 [61]. A group of matrix metalloproteinases also influences M1/M2 polarization of macrophages [62]. However, our study did not investigate the association of MMP-9 with M1/M2 polarization in the asthma with accelerated lung function decline. Therefore, to understand the causal relationship of MMP-9 and M1/M2 macrophage activation in the development of airway remodeling in asthma, the macrophage polarization in BAL and airway tissue should be addressed in future studies. 

## 5. Conclusions

We concluded that there was an increase in MMP-9 and MMP-9/TIMP-1 in airways and alveolar macrophages from chronic asthmatics with a rapid FEV_1_ decline, despite having been regularly treated with inhaled corticosteroids. An increase in MMP-9 and MMP-9/TIMP-1 in airways or AMs could contribute to a greater decline in lung function in cases of chronic asthma and could therefore be used as an indicator of chronic airway inflammation.

## Figures and Tables

**Table 1:** Baseline characteristics of the normal subjects and asthmatics.

Clinical Features	Normal Subjects (*n* = 10)	Asthma (*n* = 13) Slow FEV_1_ Decline	Asthma (*n* = 8) Fast FEV_1_ Decline
Age, year	47.6 ± 2.8	49.2 ± 3.4	49.4 ± 3.7
Gender, F/M	5/5	5/8	4/4
FVC, L	3.4 ± 0.3	3.0 ± 0.3	3.3 ± 0.2
FVC, % pred.	95.2 ± 4.7	88.8 ± 5.7	94.0 ± 5.0
FEV_1_, L	2.6 ± 0.3	2.1 ± 0.3	2.4 ± 0.2
FEV_1_, % pred.	82.4 ± 4.5	72.2 ± 6.3	77.6 ± 4.8
FEV_1_/FVC	83.3 ± 2.7	68.7 ± 3.6	73.8 ± 4.0
Annual FEV_1_ decline, mL/year		−18.2 ± 9.7	135.8 ± 14.0 ***
PC_20_, mg/dL	> 25	1.9 ± 0.5	3.5 ± 1.0

Values are mean ± SEM; F/M, female/male; FEV_1_: forced expiratory volume in one second; FVC: forced vital capacity; % pred., percent of predicted value; L: liter; PC_20_: value of methacholine provocative concentration causing a 20% decrease in FEV_1_. *** *p* < 0.0001 compared with group A.

**Table 2:** Characteristics of bronchoalveolar lavage.

Cell Profile	Normal (*n* = 10)	Asthma (*n* = 13) Slow FEV_1_ Decline	Asthma (*n* = 8) Fast FEV_1_ Decline
Total cell count, ×10^6^ cells	11.9 ± 1.7	21.2 ± 6.6 **	34.2 ± 10.1 **
Recovered volume, %	62.9 ± 3.7	44.6 ± 5.0	47.5 ± 5.8
Cell viability, %	94.4 ± 1.4	94.1 ± 1.3	91.6 ± 2.5
AMs, %	97.5 ± 0.6	87.9 ± 2.3 **	84.3 ± 5.3 **
Lymphocytes, %	1.5 ± 0.5	8.2 ± 1.8 **	8.0 ± 3.6 **
Neutrophils, %	0.7 ± 0.2	1.4 ± 0.3	5.0 ± 1.5 **^#^
Eosinophils, %	0.2 ± 0.1	2.4 ± 1.1 *	2.6 ± 1.1 *

Abbreviation: AMs = alveolar macrophages; Values present as mean ± SEM; * *p* < 0.05, ** *p* < 0.01 compared with normal subjects. # *p* < 0.05 compared with normal subjects or asthma with a slow FEV_1_ decline.

**Table 3:** The thickness of airway structure in patients with asthma.

Airway Structure	Asthma (*n* = 13) Slow FEV_1_ Decline	Asthma (*n* = 8) Fast FEV_1_ Decline	*p* Value
Epithelium, μm	27.2 ± 6.3	23.8 ± 7.4	0.733
Basement membrane, μm	7.0 ± 0.6	15.5 ± 2.2	0.0002
Subepithelium, μm	37.8 ± 4.2	138.3 ± 12.2	<0.0001

Data expressed as mean ± SEM.
